# A Novel Plasmid Entry Exclusion System in pKPC_UVA01, a Promiscuous Conjugative Plasmid Carrying the *bla*_KPC_ Carbapenemase Gene

**DOI:** 10.1128/aac.02322-21

**Published:** 2022-03-15

**Authors:** Muhammad Kamruzzaman, Amy J. Mathers, Jonathan R. Iredell

**Affiliations:** a Centre for Infectious Diseases and Microbiology, The Westmead Institute for Medical Research, The University of Sydney, Westmead, New South Wales, Australia; b Division of Infectious Disease and International Health, Department of Medicine, University of Virginia Health System, Charlottesville, Virginia, USA; c Clinical Microbiology Laboratory, Department of Pathology, University of Virginia Health System, Charlottesville, Virginia, USA; d Westmead Hospital, Westmead, New South Wales, Australia

**Keywords:** *Enterobacterales*, antibiotic resistance, carbapenemase, conjugation, entry exclusion, plasmids

## Abstract

Conjugative plasmids are the principal mediator in the emergence and spread of antibiotic resistance genes in Enterobacterales. Plasmid entry exclusion (EEX) systems can restrict their transfer into the recipient bacteria carrying closely related plasmids. In this study, we identified and characterized a novel plasmid entry exclusion system in a carbapenem resistance plasmid pKPC_UVA01, which is responsible for widespread dissemination of the *bla*_KPC_ carbapenemase gene among Enterobacterales in the United States. The identified *eex* gene in the recipient strain of different Enterobacterales species inhibited the conjugation transfer of pKPC_UVA01 plasmids at a range of 200- to 400-fold, and this inhibition was found to be a dose-dependent function of the EEX protein in recipient cells. The C terminus truncated version of *eex* or *eex* with an early termination codon at the C terminus region alleviated the inhibition of conjugative transfer. Unlike the strict specificity of plasmid exclusion by the known EEX protein, the newly identified EEX in the recipient strain could inhibit the transfer of IncP and IncN plasmids. The *eex* gene from the plasmid pKPC_UVA01 was not required for conjugative transfer but was essential in the donor bacteria for entry exclusion of this plasmid. This was a novel function of a single protein that is essential in both donor and recipient bacteria for the entry exclusion of a plasmid. This *eex* gene is found to be distributed in multidrug resistance plasmids similar to pKPC_UVA01 in different Enterobacterales species and may contribute to the stability of this plasmid type by controlling its transfer.

## INTRODUCTION

Plasmids are extrachromosomal mobile genetic elements that can transfer between bacteria by cell-cell contact (conjugation). These conjugative plasmids are primary vehicles for the dissemination of virulence and important antibiotic resistance genes (1–3) in the Enterobacterales. The conjugation process requires cell-to-cell contact, the formation of a mating pair or bridge between two participating bacteria, and the transfer of genetic material through the mating bridge from donor to recipient bacteria (4). Successful transfer of incoming plasmids may be inhibited by the presence of an identical or very closely related plasmid in the recipient cell by plasmid-encoded surface or entry exclusion and long-term persistence of the incoming plasmid is inhibited by plasmid (replicon) incompatibility (5, 6). Plasmid incompatibility prevents the propagation of two identical plasmids in the same cell due to interference between replication and/or partitioning systems (6). Surface exclusion inhibits bacterial cell-to-cell contact, but entry exclusion inhibits the subsequent transfer of plasmids after mating pair formation (5, 7). Entry exclusion systems (EES) limit risk from lethal zygosis by preventing excessive rounds of conjugation and are thought to be a normal feature of conjugative plasmids (5). EES have been identified in self-transmissible plasmids of replicon type IncF (7, 8), IncI (9–12), IncP (13–15), IncN/IncW (16–18), IncHI1 (19), and IncA/IncC (19, 20) as well as in the mobilizable plasmid ColE1 (21), integrative and conjugative elements (ICEs) of the SXT/R391 family (22), and sex pheromone plasmids pDA1 and pCF10 of Gram-positive bacteria (23–26).

The entry exclusion gene(s) in recipient cells are responsible for transfer inhibition, and, for most plasmids, the EEX protein in the recipient cell interacts with a cognate type 4 secretion system (T4SS)-type protein in the donor cell to exclude entry of the incoming conjugative plasmid. For IncC/IncF plasmids and SXT/R391 family ICEs, the EEX protein interacts with TraG or, in the case of IncI plasmids, ExcA interacts with the TraY protein in the donor cell (8, 12, 20). In the case of the IncPα plasmid RP4, a single-entry exclusion protein TrbK in the recipient cell is sufficient for entry exclusion (13).

The emergence and global spread of carbapenem-resistant Enterobacterales (CRE) worldwide (27) has featured the dissemination of plasmid-encoded carbapenem-hydrolyzing serine β-lactamases, including the Klebsiella pneumoniae carbapenemases (KPCs). The pKPC_UVA01 plasmid was a novel plasmid, which had acquired the Tn*4401* carrying *bla*_KPC-2_ (28), first associated with an outbreak of multispecies carbapenem-resistant infections in a USA healthcare institution (28, 29). More recently, pKPC_UVA01 has been described in a range of different species and genera in multiple states across the USA (30, 31). Whole-genome sequencing of the 43,621 bp plasmid revealed replication, conjugation, partitioning, and toxin-antitoxin systems (29) but no known entry exclusion systems. In this study, we described a novel entry exclusion system in pKPC_UV01 that was essential in both donor and recipient bacteria for plasmid entry exclusion and able to inhibit the transfer of unrelated plasmid types, which are widely distributed among carbapenem and β-lactam resistance plasmids of the Enterobacterales.

## RESULTS

### Entry exclusion properties of plasmid pKPC_UVA01.

The plasmid pKPC_UVA01 is not categorized into any Inc type identified by PlasmidFinder (32) but carries a replication protein (RepA) identical to IncFII-family Rep proteins in GenBank. We replaced the antibiotic resistance transposon containing regions (∼15.0 kb) of pKPC_UVA01 with a tetracycline resistance gene to construct pJIMK94 (Table 1) and transformed this into E. coli BW25113Rf to create a recipient strain for the conjugative transfer of pKPC_UVA01 from E. coli J53. Conjugative transfer of pKPC_UVA01 is inhibited nearly 1000-fold by the presence of pJIMK94 in recipient cells (Table 2).

**TABLE 1:** Plasmids used in this study

Plasmid	Characteristics	Source/ reference
pBCSK+	High copy no. (100-150 copies/cell) cloning vector, Chloramphenicol resistance	Stratagene, USA
pGEM-T Easy	TA-cloning vector, Ampicillin resistance	Promega, USA
pBAD33_Gm	Expression vector with p15A origin of replication and l-arabinose inducible promoter upstream of the multiple cloning site; Gm^R^	Addgene, plasmid no. 65098
pKPC_UVA01	Naturally occurring *bla*_KPC-2_ carrying plasmid conferring resistance to carbapenem	29
pJIMK94	∼15.0 kb multiresistant region (MRR) with transposable elements of pKPC_UVA01 was replaced with tetracycline resistance gene	This study
pJIMK97	*trbK* with its promoter and ribosome binding sites cloned into HindIII and XbaI sites of pBCSK+	This study
pJIMK97-116CT	*trbK* with 116 bp truncated from the C-terminal region was cloned into HindIII and XbaI sites of pBCSK+	This study
pJIMK97-232CT	*trbK* with 232 bp truncated from the C-terminal region was cloned into HindIII and XbaI sites of pBCSK+	This study
pJIMK98	*trbJ* with its promoter and ribosome binding sites cloned into HindIII and XbaI sites of pBCSK+	This study
pJIMK114	*trbL* with its promoter and ribosome binding sites cloned into HindIII and XbaI sites of pBCSK+	This study
pJIMK128	*ATPase-trbJ* with its promoter and ribosome binding sites cloned into HindIII and XbaI sites of pBCSK+	This study
pJIMK122	A 3016 bp DNA containing *repA* gene and surrounding region was cloned into the pGEM-T Easy vector, Amp^R^	This study
pJIMK110	In-frame deletion of *trbJ* from pKPC_UVA01 plasmid	This study
pJIMK113	In-frame deletion of *trbL* from pKPC_UVA01 plasmid	This study
pJIMK121	In-frame deletion of *trbK* from pKPC_UVA01 plasmid	This study
pJIMK130	Replaced 2 nucleotides (GG>AA, 338-39) in the C terminus region of *trbK* and cloned into HindIII and XbaI sites of pBCSK+	This study
pJIMK131	Full-length *trbK* with its ribosome binding site cloned into HindIII and XbaI sites of pBAD33_Gm	This study
pKM200	Chloramphenicol resistant plasmid carrying lambda-red recombinase system	47
pKD4	The plasmid carrying FRT-Kn cassette	46
pCP20	Temperature-sensitive FLP helper plasmid, Amp^R^	46
R751	Naturally occurring IncP plasmid, Sulfonamide resistance	33
R46	Naturally occurring IncN plasmid, trimethoprim resistance	GB accession: AY046276.1
pJIBE401	Naturally occurring conjugative IncM plasmid from clinical isolate K. pneumoniae Kp1239; identical to pEl1573	42

**TABLE 2:** Plasmid pKPC_UVA01 conjugation inhibition in the presence of its derivative (pJIMK94) in the recipient cell and vice versa

Donor	Recipient	Conjugation frequency^*a*^	Exclusion index (EI)^*b*^
J53(pKPC_UVA01)	BW25113Rf	(1.117 ± 0.31) × 10^−4^	1
J53(pKPC_UVA01)	BW25113Rf(pJIMK94)	(9.6 ± 4.7) × 10^−8^	1160 ± 42
J53(pJIMK94)	BW25113Rf	(4.35 ± 0.66) × 10^−4^	1
J53(pJIMK94)	BW25113Rf(pKPC_UVA01)	(4.41 ± 0.56) × 10^−7^	986 ± 51
J53(pJIMK94)	BW25113Rf(pGEM-T Easy)	(2.83 ± 0.3) × 10^−4^	1
J53(pJIMK94)	BW25113Rf(pJIMK122)	(3.01 ± 0.4) × 10^−6^	94 ± 3.5

a Conjugation frequency is the ratio of transconjugants/donors. The values are the mean conjugation frequency of 5 independent conjugation experiments with standard errors.

b EI is calculated by dividing the conjugation frequency to recipient bacteria BW25113Rf without any plasmid or carrying the control plasmid pGEM-T Easy with the frequency to BW25113Rf carrying plasmid pKPC_UVA01, its derivative pJIMK94 or plasmid pJIMK122.The values are the mean of 5 independent conjugation experiments with standard errors.

To eliminate any effect from replicon-based incompatibility, a 3016 bp region from pKPC_UVA01, which includes the pKPC_UVA01 replication gene *repA* with 1424 bp upstream and 978 bp downstream sequences was cloned in a cloning vector pGEM-T Easy (Phage f1 replicon) to construct pJIMK122 (AmpR). pJIMK122 and pKPC_UVA01 were then transferred separately into E. coli BW25113Rf. Transfer of pJIMK94 (Tet^R^ derivative of pKPC_UVA01) from donor J53 to recipient BW25113Rf carrying pKPC_UVA01 was ∼10× less efficient (∼10× more inhibited) than into BW25113Rf carrying pJIMK122, suggesting an inhibition mechanism beyond that of the recognized plasmid incompatibility (Table 2).

### Identification of a putative *trbK* entry exclusion gene.

**FIG 1:**
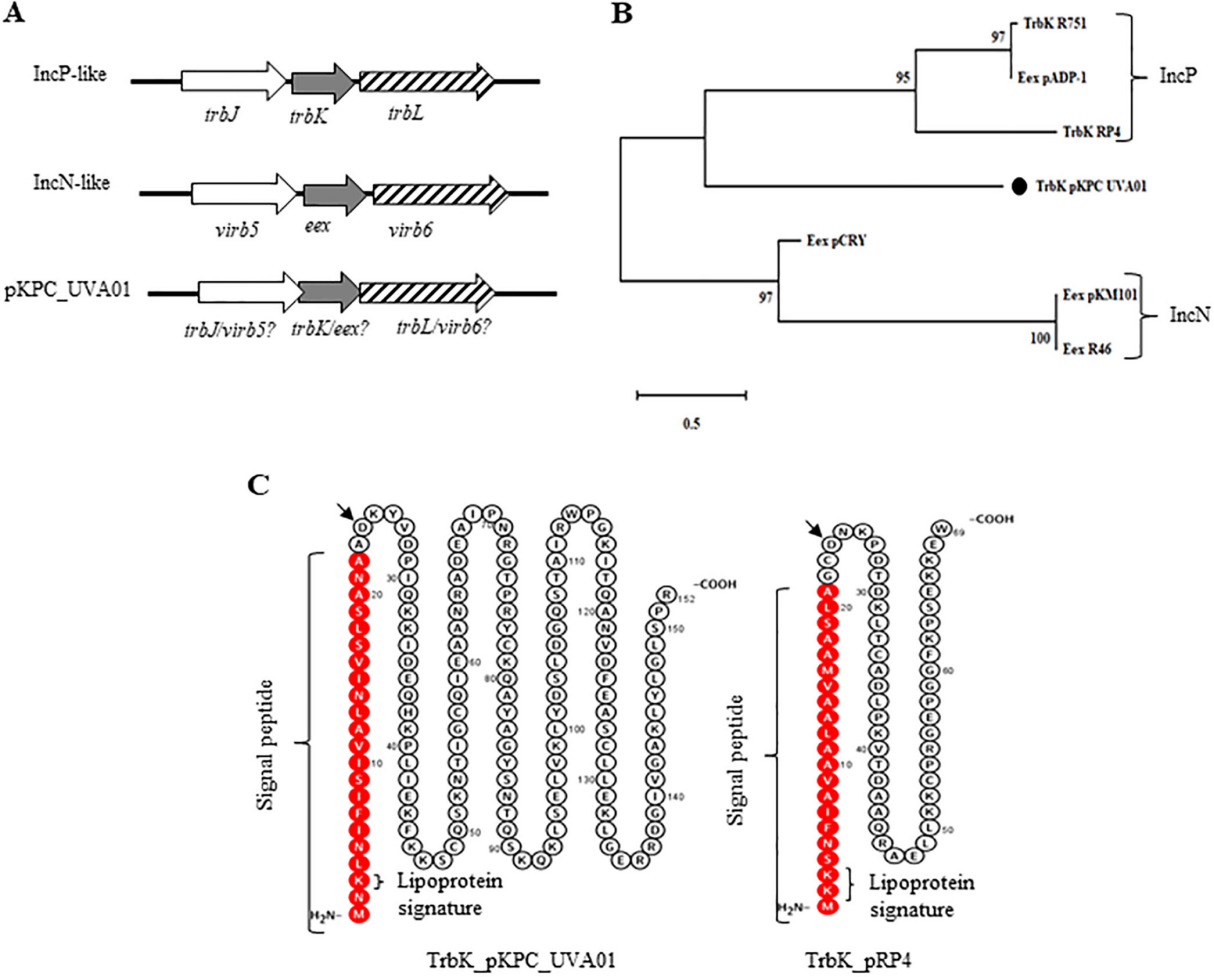
(A) Comparison of the genetic context of putative entry exclusion gene of plasmid pKPC_UVA01 with that of IncP plasmid RP4 and IncN plasmid R46. Entry exclusion genes are filled in gray. (B) Amino acid sequences of TrbK from pKPC_UVA01 and some other entry exclusion protein of known IncP and IncN plasmids were aligned with ClustalW of MEGA X then phylogenetic tree for the genetic relatedness of the entry exclusion proteins (EEX) was constructed by the maximum likelihood method of MEGA X with a bootstrap value of 500. The percentage of trees in which the associated proteins clustered together is shown next to the branches. TrbK entry exclusion protein of pKPC_UVA01 is indicated with a black circle. (C) Analysis of the protein sequences of EEX protein of pKPC_UVA01 and IncP plasmid RP4. Predicted signal peptide sequences are indicated in red, positively charged amino acids for lipoprotein signature are shown, and conserved aspartate amino acid residue is indicated by the arrow.

EEX systems are typically located in the plasmid transfer regions (5). For example, the exclusion gene *trbK* in IncP plasmid is surrounded by *trbJ* and *trbL* and the *eex* gene in IncN plasmid is surrounded by *virb5* and *virb6* like genes upstream and downstream, respectively (Fig. 1A). A *trbL/virb6-*like gene can be identified in pKPC_UVA01 (Fig. 1A), upstream of which were two genes of unknown functions, including one most immediately upstream of *trbL* with 35%, 28%, and 30% amino acid sequence identity with TrbK of IncP plasmid RP4 (13), TrbK of IncP plasmid R751 (33) and with EEX of IncN plasmid R46 (17, 34), respectively (Fig. S1). Further upstream of this *trbK* homolog is a gene encoding a hypothetical protein with no match to known conjugation transfer proteins in GenBank but with 22% amino acid sequence identity with TrbJ of RP4 and 41% identity with a type 6 secretion protein of Salmonella and E. coli (unpublished data). We considered the *trbK* homolog of pKPC_UVA01 as a candidate entry exclusion gene for pKPC_UVA01 and use *trbJ*, *trbK,* and *trbL* to refer to the three putative EES genes (Fig. 1A) of plasmid pKPC_UVA01 in the remainder of the manuscript. The putative *trbK* exclusion gene of pKPC_UVA01 has a similar genetic context to both IncP and IncN plasmid entry exclusion systems (Fig. 1A) and amino acid sequence alignment and phylogenetic analysis of TrbK from pKPC_UVA01 with TrbK, and EEX proteins of several IncP and IncN plasmids suggests a distant relationship (Fig. 1B).

TrbK of IncP plasmid RP4 was 69 aa long while TrbK of pKPC_UVA01 was predicted to be twice this size at 151 aa long. However, both had N-terminal lipoprotein targeting signal peptides (Fig. 1C) as predicted by the online protein analysis tool Protter (http://wlab.ethz.ch/protter) and were predicted to be localized in the cytoplasmic membrane by protein subcellular localization prediction tool Cello (http://cello.life.nctu.edu.tw), which is similar to the TrbK_IncP. Protein sequence analysis confirmed that both TrbKs had a conserved aspartate residue at the position +2 in the mature polypeptide, suggesting their localization in the cytoplasmic membrane (35). The aspartate amino acid at this position was thought to function as an assorting signal for lipoprotein localization (36, 37).

### The *trbK* gene of pKPC_UVA01 is the plasmid entry exclusion gene in recipient cells.

To examine the entry exclusion function encoded by the *trbK* gene of pKPC_UVA01, the full-length coding region of *trbK* gene with its putative promoter and ribosome binding site was cloned into a cloning vector pBCSK+ (ColE1 replicon) to construct pJIMK97 (Table 1). Conjugative transfer of pKPC_UVA01 from E. coli J53 was greatly inhibited by the presence of pJIMK97 (carrying pKPC_UVA01-derived *trbK*) in the recipient strain E. coli BW25113Rf (Table 3) with an exclusion index (EI) >300 compared to the empty recipient or recipient carrying control vector pBCSK+. Transfer inhibition of pKPC_UVA01 from E. coli J53 into K. pneumoniae was also strongly inhibited by pJIMK97 (pKPC_UVA01-derived *trbK*) with an exclusion index of ∼400 (Table 3).

**TABLE 3:** TrbK in the recipient cell strongly inhibits the conjugation of pKPC_UVA01, and C-terminal truncation or a premature stop codon at the C terminus eliminates the inhibition effect

Donor	Recipient	Conjugation frequency^*a*^	Exclusion index (EI)^*b*^
J53(pKPC_UVA01)	BW25113Rf	(3.15 ± 0.34) × 10^−5^	1
J53(pKPC_UVA01)	BW25113Rf (pBCSK+)	(2.35 ± 0.39) × 10^−5^	1.38 ± 0.33
J53(pKPC_UVA01)	BW25113Rf (pJIMK97)	(7.82 ± 1.5) × 10^−8^	302 ± 19
J53(pKPC_UVA01)	Kp ATCC 13883Rf	(8.16 ± 0.25) × 10^−6^	1
J53(pKPC_UVA01)	Kp ATCC 13883Rf (pBCSK+)	(5.66 ± 0.27) × 10^−6^	1.4 ± 0.07
J53(pKPC_UVA01)	Kp ATCC 13883Rf (pJIMK97)	(1.52 ± 0.13) × 10^−8^	378 ± 15
J53(pKPC_UVA01)	BW25113Rf (pJIMK97-116CT)	(1.4 ± 0.21) × 10^−5^	1.7 ± 0.12
J53(pKPC_UVA01)	BW25113Rf (pJIMK97-232CT)	(1.11 ± 0.25) × 10^−5^	2 ± 0.39
J53(pKPC_UVA01)	BW25113Rf (pJIMK130)	(1.28 ± 0.39) × 10^−5^	2.0 ± 0.63

a Conjugation frequency is the ratio of transconjugants/donors. The values are the mean conjugation frequency of 8 independent conjugation experiments with standard errors.

b EI is calculated by dividing the conjugation frequency of pKPC_UVA01 to recipient bacteria BW25113Rf without any plasmid or carrying the control plasmid pBCSK+ with the frequency to BW25113Rf carrying cloned *trbK* or its truncated versions or *trbK* with a premature stop codon at C terminus. The values are the mean of 8 independent conjugation experiments with standard errors.

### The C-terminal region of *trbK* is essential for entry exclusion.

Two versions of the plasmid carrying pKPC_UVA01-derived *trbK* were constructed to examine the role of the C-terminal region of TrbK in entry exclusion. A 116 and 232 bp truncated versions from the C terminus of *trbK* gene with the same promoter and RBS were cloned into the pBCSK+ cloning vector as pJIMK97-116CT and pJIMK97-232CT, respectively (Table 1). E. coli strain BW25113Rf was transformed with 2 of these plasmids separately and used as a recipient for the conjugative transfer of pKPC_UVA01 plasmid from donor J53 strain. Neither of the C termini truncated versions of *trbK* inhibited the transfer of incoming pKPC_UVA01 (Table 3), suggesting that the C-terminal region of TrbK is essential for entry exclusion. To further confirm the role of the C-terminal region of *trbK* in plasmid entry exclusion, we synthesized a full-length *trbK* (the same *trbK* region of pJIMK97) with an early termination codon at the C-terminal region (Fig. S2) and cloned it into the pBCSK+ and constructed pJIMK130 (Table 1). No conjugation transfer inhibition of pKPC_UVA01 from J53 to BW25113Rf(pJIMK130) was observed in comparison to the empty BW25113Rf recipient (Table 3). Moreover, we examined whether there were any changes in the *trbK* expression from the constructs with intact (pJIMK97), truncated (pJIMK97-116CT), or mutated (pJIMK130) *trbK*. The relative expression of *trbK* was very similar to all the constructs examined (Fig. S3) by quantitative real-time PCR assay (qRT PCR). These data fully confirmed that the C-terminal region of TrbK is essential in the recipient cell to inhibit the transfer of pKPC_UVA01.

### TrbK dosage effects.

To examine gene dosage effects, the full-length *trbK* with its RBS was cloned into the expression vector pBAD33_Gm under the control of an arabinose inducible promoter, to construct pJIMK131 (Table 1). Relative expression of *trbK* from E. coli BW25113Rf(pJIMK131) was directly related to the level of induction with different concentrations of arabinose (0%, 0.001%, 0.01%, and 0.04%) (Table 4, Fig. S4A). We then examined the transfer of pKPC_UVA01 from the J53 donor to the recipient BW25113Rf(pJIMK131) after induction with different concentrations of arabinose (mentioned earlier). The conjugation transfer of pKPC_UVA01 was strongly inhibited with the increased expression of *trbK* from pJIMK131 in the recipient bacteria (Table 4, Fig. S4). A clear dose-effect was evident with an exclusion index as high as >2800 when *trbK* was induced most strongly (0.04% arabinose).

**TABLE 4:** Dosages of TrbK in recipient bacteria influence the conjugation of pKPC_UVA01 from donor bacteria

Donor	Recipient	Arabinose dosages (%)	Relative TrbK expression (fold change)	Conjugation frequency^*a*^	Exclusion index (EI)^*b*^
J53(pKPC_UVA01)	BW25113Rf(pJIMK131)	0	3.3 ± 0.57	(9.57 ± 0.58) × 10^−5^	1
J53(pKPC_UVA01)	BW25113Rf(pJIMK131)	0.001	49.3 ± 3.5	(2.67 ± 0.58) × 10^−7^	358 ± 18
J53(pKPC_UVA01)	BW25113Rf(pJIMK131)	0.01	220.3 ± 10.59	(8.57 ± 0.25) × 10^−8^	1117 ± 119
J53(pKPC_UVA01)	BW25113Rf(pJIMK131)	0.04	388 ± 21	(3.37 ± 0.41) × 10^−8^	2840 ± 189

a Conjugation frequency is the ratio of transconjugants/donors. The values are the mean conjugation frequency of 3 independent conjugation experiments with standard errors.

b EI is calculated by dividing the conjugation frequency to recipient bacteria BW25113Rf (pJIMK131) without arabinose with the frequency to BW25113Rf(pJIMK131) with arabinose. The values are the mean of 3 independent conjugation experiments with standard errors.

### *trbJ* and *trbL* but not *trbK* were required for the conjugative transfer of pKPC_UVA01.

For entry exclusion, plasmid-encoded EEX protein in the recipient cell interacts with plasmid-encoded proteins in the donor cell. In most well-characterized plasmid entry exclusion systems, the donor’s cognate exclusion factor is usually located immediately upstream or downstream of the exclusion gene in the plasmid. For example, the exclusion factors TraG (in IncC and IncF plasmids) and TraY (IncL/M plasmids) are encoded from genes immediately upstream of the exclusion gene *eex* (5). Genetic structure analysis of the pKPC_UVA01 exclusion gene and its surrounding regions (Fig. 1A) indicated a *trbL* gene downstream of *trbK*, which has 99% protein sequence identity with the VirB8 protein of the type 6 secretion system of *Citrobacter* spp. The *trbK* gene has a single nucleotide overlap with the immediate upstream gene *trbJ*. The *trbJ* had four nucleotides that overlap an upstream gene *AAA ATPases*. To determine whether these three genes (*ATPase*, *trbJ,* and *trbK*) were expressed from a common promoter, we searched for promoter sequences and RBS in the upstream region of these genes. Prokaryotic consensus RBS sequences were found immediately upstream of all three genes and, strong promoters were predicted upstream of *ATPase* and *trbK* with only a weak promoter predicted for *trbJ* by the online promoter prediction tool BPROM (http://www.softberry.com/berry.phtml?topic=bprom&group=programs&subgroup=gfindb). This suggested that *trbK* may be individually expressed but *trbJ* may rely on transcriptional linkage with the *ATPase* gene. In-frame deletion mutants for *trbL*, *trbJ*, and *trbK* genes from pKPC_UVA01 were, therefore, constructed (Table 1) and transformed into E. coli J53 strain.

The deletion of the exclusion gene *trb*K from pKPC_UVA01 did not interfere in the conjugation transfer of the plasmid into empty BW25113Rf (Table 5). However, in-frame deletion of *trbL* or *trbJ* completely abolished conjugative transfer to empty recipient E. coli BW25113Rf (Table 5). A *trbL* deletion was fully complemented from a cloning vector in *trans* (Table 1) and *ATPase-trbJ*, but not *trbJ*, complemented a Δ*trbJ* in-frame deletion to restore conjugation of pKPC_UVA01 (Table 5), confirming TrbJ and TrbL to be essential proteins for conjugation of pKPC_UVA01 and suggesting that *trbJ* relied primarily on a transcriptional read-through of the upstream *AAA ATPase* gene.

**TABLE 5:** Role of the exclusion gene *trbK* and its surrounding genes *trbJ* and *trbL* in the conjugation transfer of the plasmid pKPC_UVA01

Donor	Recipient	Transconjugants	Conjugation frequency^*a*^
J53(pKPC_UVA01)	BW25113Rf	(1.8 ± 0.45) × 10^5^	(4.0 ± 1.2) × 10^−4^
J53(pKPC_UVA01Δ*trbJ*)	BW25113Rf	0	0
J53(pKPC_UVA01Δ*trbJ* + pBCSK+)	BW25113Rf	0	0
J53(pKPC_UVA01Δ*trbJ* + *trbJ* in pBCSK+)	BW25113Rf	0	0
J53(pKPC_UVA01Δ*trbJ* + *ATPase*-*trbJ* in pBCSK+)	BW25113Rf	(7.0 ± 1.54) × 10^4^	(1.4 ± 0.23) × 10^−4^
J53(pKPC_UVA01Δ*trbL*)	BW25113Rf	0	0
J53(pKPC_UVA01Δ*trbL* +pBCSK+)	BW25113Rf	0	0
J53(pKPC_UVA01Δ*trbL* + *trbL* in pBCSK+)	BW25113Rf	(1.1 ± 0.21) × 10^5^	(2.2 ± 0.34) × 10^−4^
J53(pKPC_UVA01Δ*trbK*)	BW25113Rf	(3.0 ± 0.47) × 10^4^	(9.3 ± 1.2) × 10^−5^

a Conjugation frequency is the ratio of transconjugants/donors. The values are the mean conjugation frequency of 3 independent conjugation experiments with standard errors.

### Entry exclusion by TrbK was dependent on the presence of *trbK* in the donor cell.

We showed that *trbK* of pKPC_UVA01 was not required for conjugative transfer but was essential for entry exclusion by the recipient cell. Interestingly, however, the Δ*trbK* mutant of pKPC_UVA01 (pJIMK121) was readily received by bacteria carrying cloned *trbK* (pJIMK97, which powerfully exclude the native pKPC_UVA01) at rates comparable to empty recipient bacteria (Table 6). These data implied that TrbK was essential in both donor and recipient bacteria and had a dual role in the entry exclusion of plasmid pKPC_UVA01.

**TABLE 6:** Entry exclusion of pKPC_UVA01 is dependent on the presence of *trbK* in the donor bacteria

Donor	Recipient	Conjugation frequency^*a*^	Exclusion index (EI)^*b*^
J53(pKPC_UVA01)	BW25113Rf	(2.0 ± 0.51) × 10^−5^	1
J53(pKPC_UVA01Δ*trbK*)	BW25113Rf	(9.3 ± 1.23) × 10^−6^	2.1 ± 0.56
J53(pKPC_UVA01)	BW25113Rf(pJIMK97)	(6.3 ± 0.98) × 10^−8^	317 ± 31
J53(pKPC_UVA01Δ*trbK*)	BW25113Rf(pJIMK97)	(3.88 ± 0.66) × 10^−6^	5 ± 0.91
J53(pKPC_UVA01Δ*trbK*)	BW25113Rf(pJIMK97-116CT)	(1.25 ± 0.32) × 10^−5^	1.6 ± 0.25

a Conjugation frequency is the ratio of transconjugants/donors. The values are the mean conjugation frequency of 5 independent conjugation experiments with standard errors.

b EI is calculated by dividing the conjugation frequency to empty recipient bacteria BW25113Rf with the frequency to BW25113Rf carrying plasmid pJIMK97 or its truncated version. The values are the mean of 5 independent conjugation experiments with standard errors.

### TrbK of pKPC_UVA01 excluded entry of IncP and IncN plasmids.

We showed that TrbK of pKPC_UVA01 had a significant protein-level identity with the entry exclusion protein TrbK of IncP plasmid and EEX protein of IncN plasmid predicted to be much larger than other TrbK-like proteins and appeared to have a dual function. We, therefore, measured the transfer of an IncP plasmid, R751, and an IncN plasmid, R46 from E. coli J53 to E. coli BW25113Rf carrying pJIMK94 (Table 1, the ΔMRR derivative of pKPC_UVA01 with complete entry exclusion apparatus) or cloned pKPC_UVA01-derived *trbK* (pJIMK97). Plasmid-free BW25113Rf and BW25113Rf carrying an IncM plasmid (pJIBE401 with an unrelated entry exclusion system) were used as controls. Interestingly, pKPC_UVA01-derived *trbK* (from both pJIMK97, cloned *trbK*, and pJIMK94, a derivative of the natural plasmid) inhibited the incoming transfer of both IncP and IncN plasmids (Table 7) with an exclusion index between 50 to 90, suggesting that the entry exclusion protein TrbK of pKPC_UVA01 had an extended range of exclusion activity that included relatively unrelated plasmids. We did not identify any transfer inhibition of plasmid pKPC_UVA01 in the recipient bacteria carrying either IncN plasmid R46 or IncP plasmid R751 (Table 7).

**TABLE 7:** Entry exclusion of an IncP and an IncN plasmid by the exclusion protein TrbK of pKPC_UVA01 and vice versa

Donor	Recipient	Conjugation frequency^*a*^	Exclusion index (EI)^*b*^
J53(IncP plasmid R751)	BW25113Rf	(2.6 ± 0.6) × 10^−3^	1
J53(IncP plasmid R751)	BW25113Rf(pJIBE401)	(1.5 ± 0.67) × 10^−3^	1.7 ± 0.53
J53(IncP plasmid R751)	BW25113Rf(pJIMK94)	(4.6 ± 0.25) × 10^−5^	56 ± 9
J53(IncP plasmid R751)	BW25113Rf(pJIMK97)	(3.8 ± 0.53) × 10^−5^	68 ± 7
J53(IncN plasmid R46)	BW25113Rf	(1.3 ± 0.36) × 10^−2^	1
J53(IncN plasmid R46)	BW25113Rf(pJIBE401)	(1.6 ± 0.56) × 10^−2^	0.80 ± 0.15
J53(IncN plasmid R46)	BW25113Rf(pJIMK94)	(2.2 ± 0.50) × 10^−4^	59 ± 4
J53(IncN plasmid R46)	BW25113Rf(pJIMK97)	(1.5 ± 0.55) × 10^−4^	86 ± 13
J53(pKPC_UVA01)	BW25113Rf	(2.5 ± 0.44) × 10^−5^	1
J53(pKPC_UVA01)	BW25113Rf(R751)	(1.7 ± 0.50) × 10^−5^	1.5 ± 0.56
J53(pKPC_UVA01)	BW25113Rf(R46)	(2.8 ± 0.53) × 10^−5^	0.89 ± 0.05

a Conjugation frequency is the ratio of transconjugants/donors. The values are the mean conjugation frequency of 3 independent conjugation experiments with standard errors.

b EI is calculated by dividing the conjugation frequency to empty recipient bacteria BW25113Rf with the frequency to BW25113Rf carrying plasmid pJIBE401 or pJIMK97 or pJIMK94 or R751 or R46. The values are the mean of 3 independent conjugation experiments with standard errors.

### Distribution of *trbK* in GenBank.

**FIG 2:**
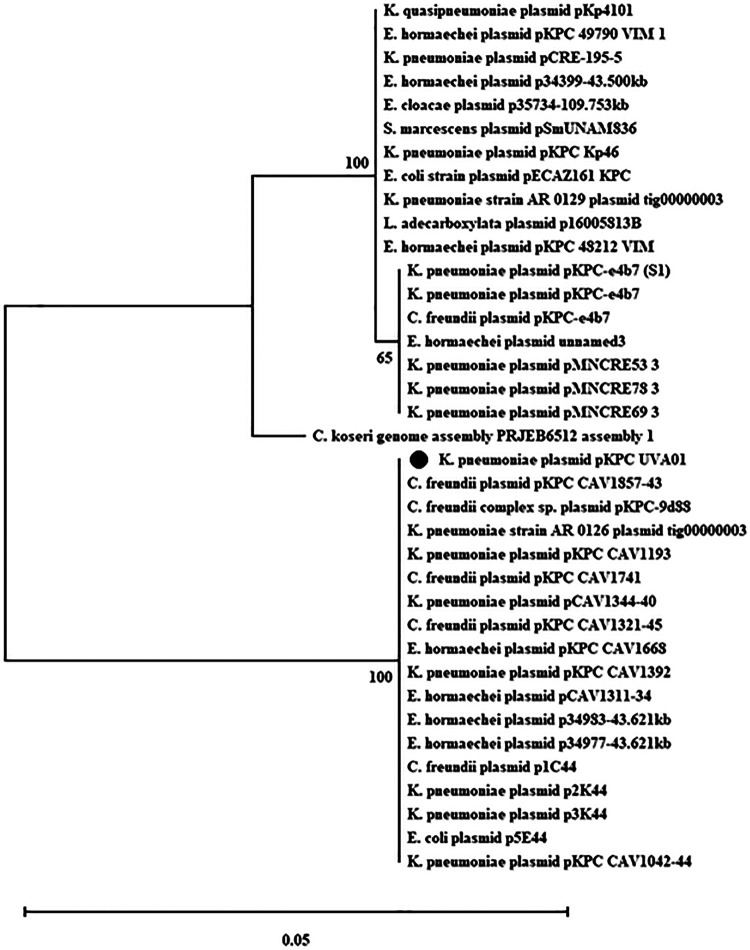
Distribution of the pKPC_UVA01 entry exclusion gene (*trbK*) in the GenBank and phylogenetic tree for the genetic relatedness with the *trbK* homologs found in the plasmids of Enterobacterales was constructed by the Maximum Likelihood Method of MEGA X with a bootstrap value of 500. The percentage of trees in which the associated proteins clustered together is shown next to the branches. TrbK of pKPC_UVA01 is indicated with a black circle.

A nucleotide blast (BLASTN) search for the full-length *trbK* gene yielded 37 hits in the GenBank (Table S1) with 100% nucleotide sequence length coverage and 93 to 100% nucleotide sequence identity. All were found on plasmids from Enterobacterales, mostly Klebsiella, Citrobacter, and Enterobacter species (Fig. 2). Almost all ranged from 43 to 55 kb in size, which was similar to pKPC_UVA01 (43 kb) with three large (109, 129, and 187 kb) and one small (∼26 kb) plasmid (Table S1). PlasmidFinder (32) annotated these with the same replication gene/system, pKPC_CAV1193 replicon, which was not an incompatibility type identified by PCR-based replicon typing (PBRT) schemes but came from the same hospital as the originally identified pKPC_UVA01 and, therefore, was designated a pKPC_UVA01 type replicon (instead of pKPC_CAV1193) in Table S1. Three plasmids had an additional replicon (IncM1, IncC, and ColRNAI; Table S1), two of these were large (129 and 187 kb) and one a similar in size to the pKPC_UVA01 plasmid (55 kb). ResFinder (32) analysis of plasmids carrying *trbK* genes revealed that all except one plasmid carry antibiotic resistance genes (Table S1) with most (32/37) as carbapenemase genes (mostly KPC type: 21 with *bla*_KPC-2_, 8 with *bla*_KPC-3_, 2 with *bla*_VIM-1,_ and one with *bla*_IMP-8_). Most of these plasmids had *bla*_TEM_ genes, a few had *bla*_OXA,_ and one had *bla*_CMY-4_ genes. The newly identified entry exclusion gene *trbK* was distributed among plasmids carrying clinically important antimicrobial resistance (AMR).

## DISCUSSION

Plasmid entry exclusion benefits both bacteria and plasmids, reducing unnecessary conjugative transfer events that consume energy in the donor and damage the recipient (5). Most, or perhaps all, conjugative plasmids have entry exclusion systems, but these tend to be conserved and specific in each plasmid Inc type. Although functionally characterized in a few well-known plasmid types, they remain largely unknown for most. Here, we identified and characterized an entry exclusion system in the conjugative plasmid pKPC_UVA01, carrier of the *bla*_KPC_ carbapenemase gene in many different species of the Enterobacterales and responsible for major outbreaks in USA health care systems (29, 31, 38). The identified entry exclusion protein has amino acid sequence, structural and functional identity with the TrbK (EEX protein) of IncP plasmid RP4 (13) with a predicted N-terminal signal peptide (39) characterized by positively charged lipoprotein signature and hydrophobic core region (Fig. 1C).

We showed that the TrbK_pKPC_UVA01 C-terminal region was essential for exclusion functions, as was previously shown for TrbK_IncP (13). TrbK_pKPC_UVA01 strongly inhibited the transfer of pKPC_UVA01 into different Enterobacterales species with an exclusion index in the range of 200 to 400 that seems to vary with recipient bacterial species and with TrbK dosage, as also previously found for TrbK_IncP (14). For most plasmids, exclusion requires distinct plasmid-encoded factors are expressed in both donor and recipient cells (8, 12, 20). No such donor factor has been identified as a cognate TrbK partner in the well-studied IncP plasmids, and for entry exclusion, IncP TrbK functions only in the recipient cell (13). Here, we showed that a single protein from pKPC_UVA01, TrbK, provides the cognate entry exclusion requirements of both donor and recipient cells. It was previously reported that entry exclusion genes are necessary for both donor and recipient bacteria to exhibit entry exclusion of the IncHI1 plasmid R27 (19). While *trbK* may provide dual-factor exclusion of pKPC_UVA01, it was not essential for the conjugative transfer of this plasmid. Two surrounding genes were found to be required (Table 5). The genes encoding plasmid entry exclusion functions are in the conjugation transfer regions but were shown to be nonessential for transferring those plasmids (9, 14, 18, 40). For example, the closely related entry exclusion protein TrbK of IncP plasmid RP4 is not essential for RP4 specific transfer or mobilization of a nonconjugative mobilizable IncQ plasmid (14).

TrbK_pKPC_UVA01 has 28 to 35% amino acid sequence identity with IncP and IncN exclusion proteins, and the phylogenetic tree suggests a closer link to TrbK_IncP plasmids (Fig. 1B). Exclusion is generally specific, and TrbK_IncP is reported not to interfere with the transfer of unrelated plasmids (13) being specific to IncP plasmid types. Even the TrbK of IncPα plasmid RP4 has higher exclusion efficiency for the IncPα plasmid than IncPβ plasmid R751, and TrbK of these two plasmids has 41% identity (13). The plasmid replication system of pKPC_UVA01 is quite different from that of the IncP or IncN plasmids, and the exclusion proteins were <35% identical, so the cross-inhibition of IncP or IncN plasmid transfer by TrbK_pKPC_UVA01 was unexpected. However, interestingly, it was found that entry exclusion protein TrbK from pKPC_UVA01 in the recipient cell can inhibit the entry of unrelated IncP and IncN plasmids, suggesting a broad-spectrum entry exclusion function. Two groups of compatible plasmids, IncC and IncA, also showed strong mutual entry exclusion by closely related entry exclusion systems (20, 41).

An effective entry exclusion system, promoting plasmid stability and protecting host bacteria from lethal zygosis, is to be expected in successful plasmids such as pKPC_UVA01, a major vehicle for important AMR genes in the Enterobacterales species (28, 29). The wide distribution of this *trbK* entry exclusion gene in AMR plasmids among Enterobacteriales species indicates its significant value, to which a unique dual functionality and unusually broad action against other plasmids may add.

The identification and characterization of this entry exclusion gene may also help to design control strategies for the spread of AMR plasmids (42). Manipulation of entry exclusion may improve the transfer efficiency of conjugative curing plasmids.

We characterized an entry exclusion system in a clinically and epidemiologically important novel plasmid responsible for the spread of *bla*_KPC_ in the United States. This work provides insight into the basic mechanisms of horizontal gene transfer by important AMR plasmids related to pKPC_UVA01. Further understanding of plasmid transfer mechanisms will likely be central to slowing the continued spread of antimicrobial resistance within Enterobacterales.

## MATERIALS AND METHODS

### Bacterial strains, plasmids, and primers.

Escherichia coli DH10B (catalog number EC0113, Thermo Fisher Scientific) was used as a host for cloning and recombinant vectors. Sodium azide resistance E. coli J53 (42), rifampicin resistance E. coli BW25113Rf (43), and rifampicin resistance Klebsiella pneumoniae strain ATCC 13883Rf (42) were used in plasmid conjugation experiments. Plasmids and primers used in this study were described in Table 1 and Table S2, respectively. Bacteria were grown in LB broth/agar at 37°C, and bacteria carrying plasmids were grown with appropriate antibiotics in culture media. CHROMagar^TM^ orientation with appropriate antibiotics was used to select transconjugants. Antibiotics ampicillin (75 μg/mL), chloramphenicol (20 μg/mL), kanamycin (50 μg/mL), tetracycline (10 μg/mL), sodium azide (100 μg/mL), and rifampicin (90 μg/mL) were used.

### Bioinformatics.

BLASTP search was performed for each protein sequence presented in the conjugative transfer region of plasmid pKPC_UVA01 to examine their identity with the known protein sequences in the GenBank. BLASTP searches were performed using the amino acid sequences of different open reading frames (ORFs) as separate inputs against nonredundant protein sequence database and reference proteins sequence database with expectation values (E value) less than or equal to 0.01 with coverage ≥80%. Amino acid sequence alignment of two or more protein sequences was performed by using the ClustalW alignment of MEGA X software (44) using default parameters.

BLASTN search was used to determine the distribution of *trbK*_pKPC_UVA01 to the GenBank sequences using parameters described previously (45). Then the phylogenetic tree was constructed for genetic distances between nucleotide sequences identified in the GenBank sequences by MEGA X software. The evolutionary history was inferred using the maximum likelihood method. Initial trees for the heuristic search were obtained automatically by applying the Neighbor-Join and BioNJ algorithms to a matrix of pairwise distances estimated using a JTT model, then selecting the topology with a superior log-likelihood value with a bootstrap value of 500.

Protein structure analysis for the presence of signal peptide and lipoprotein signature was predicted using the online interactive protein visualization tool Protter (http://wlab.ethz.ch/protter). Localization of the protein in the cell was predicted using the online protein subcellular prediction tool Cello (http://cello.life.nctu.edu.tw).

### Construction of recombinant plasmids.

Individual genes *trbK*, *trbJ,* and *trbL* with their respective promoter and ribosome binding sites (RBS) from pKPC_UVA01 were cloned into the HindIII and XbaI restriction sites of cloning vector pBCSK+ to construct pJIMK97, pJIMK98, and pJIMK114, respectively. pJIMK97-116CT and pJIMK97-232CT were constructed by cloning *trbK* with 116 and 232 bp truncated versions from the C terminus region. The *trbJ* gene of pKPC_UVA01was overlapped with the upstream gene *AAA-ATPase* and may express as an operon. Therefore, *ATPase-trbJ* gene was cloned with their common promoter and RBS into HindIII and XbaI sites of pBCSK+ to construct pJIMK128 (Table 1). All the constructs were confirmed by PCR and verified by Sanger sequencing.

A gBlock of *trbK* with an early termination codon (GG>AA, at position 338 to 339 of coding region) near the C-terminal region (Fig. S2) was synthesized from Integrated DNA Technologies (IDT Inc.). We have designed the *trbK* gBlock with HindIII and XbaI restriction sites, and the nucleotide sequence is the same as *trbK* genetic region cloned in pJIMK97, except the early termination codon. The gBlock DNA was digested with HindIII and XbaI, and purified product was cloned into the pBCSK+ in respective restriction sites to construct pJIMK130 (Table 1).

A full-length *trbK* with its RBS was amplified with TrbK_F_EcoRI and TrbK_R_XbaI (Table S2) and restriction digested, and purified DNA was cloned into expression vector pBAD33_Gm under the arabinose inducible promoter to construct pJIMK131 (Table 1).

### Construction of gene knockout mutants.

The in-frame deletion of each gene *trbk*, *trbJ*, and *trbL* was performed by homologous recombination-based allelic exchange method, mediated by lambda-red recombinase system (46, 47). Briefly, a FRT-kanamycin cassette was amplified from the plasmid pKD4 (46) with the primers P1 and P2 carrying the 40 bp homologous regions from the upstream and downstream of the desired deletion site. The amplified PCR products were then purified by a PCR purification kit. Greater than 1.0 μg of the purified PCR product was electroporated into the electrocompetent E. coli J53 bacteria carrying plasmid pKPC_UVA01 and lambda-red recombinase plasmid pMK200 (47). The transformants with desired gene knockout were selected on agar plates with kanamycin (KAN). A few colonies were tested for gene-specific PCR to confirm the deletion. The temperature-sensitive lambda-red plasmid will be lost at 37 to 42°C. The KAN cassette was removed from the in-frame deletion mutant by introducing plasmid pCP20. Kanamycin-resistant (Km^R^) mutants were transformed with pCP20, and ampicillin-resistant transformants were selected at 30°C. A few colonies were grown at 43°C and then tested for loss of all antibiotic resistance. The majority lost the FRT-flanked KAN resistance gene and the FLP helper plasmid simultaneously (46).

Plasmid pJIMK94, the derivative of pKPC_UVA01 plasmid, was constructed by deleting ∼15.0 kb multiresistant region and transposable elements with tetracycline resistance gene by using primers (AbR_nest_F1, F2, R1, R2, listed in Table S2). *tetA* gene-specific primers with 80 bp homologous sequence of the target region for deletion were used, and this was achieved by two rounds of nested PCR.

### Plasmid conjugation frequency measurement.

Plasmid transfer rates by conjugation were measured by solid mating on membrane filters (48). For the mating experiment, donor and recipient cells were each grown overnight at 37°C in LB broth with appropriate antibiotics. The cultures were then centrifuged and washed with an equivalent volume of sterile saline solution (0.85% NaCl) and resuspended in saline. For arabinose induction, the recipient BW25113Rf(pJIMK131) bacteria were induced for one additional hour with different concentrations of arabinose (0%, 0.001%, 0.01% and 0.04%). The optical density at 600 nm (OD_600_) of each culture was adjusted to 1.0, 1 mL of each of the donor and recipient cultures were mixed, centrifuged, and the pellets were resuspended in 50 μL saline. The mixture was then spotted onto nitrocellulose membranes (GE Healthcare, IL, USA) on LB agar plates. Mating mixtures were incubated at 37°C for 4 h, and the mating filters were placed in Falcon tube in 5 mL saline followed by vortexing until the pellet was dissolved. Donor cell numbers were determined by plating dilutions of the OD_600_-adjusted donor cultures onto CHROMagar plates. The number of transconjugants was determined by plating dilutions of the mating mixtures onto CHROMagar plates containing the appropriate antibiotics. Conjugation frequencies were calculated by dividing the number of transconjugants by the number of original donor cells. The ratio of conjugation inhibition of plasmid from donor bacteria to two competitor recipient bacteria has been termed “exclusion index (EI)” and was measured by dividing the conjugation frequency of two compared groups.

### Quantitative real-time PCR (qRT-PCR).

Stationary-phase cultures of E. coli BW25113Rf bearing the pJIMK131 (*trbK* in pBAD33_Gm) plasmid were diluted 1:50 in 20 mL gentamicin-containing (8 μg/mL) LB medium and incubated with shaking at 37°C. After approximately 3 h growth, culture was divided into 4 tubes (5 mL in each) and treated with different concentrations of arabinose (0%, 0.001%, 0.01%, and 0.04%). The arabinose-induced cultures were grown for 1 h and harvested by centrifugation at 10,000 rpm for 10 min. BW25113Rf strain with other *trbk* constructs (pJIMK97, pJIK97_116CT, and pJIMK130) were grown into a stationary-phase, and 1 mL of each culture was harvested by centrifugation at 10,000 rpm for 10 min.

Bacterial pellets were treated with 400 μg/mL lysozyme in TE (10 mM Tris-Cl and 1 mM EDTA) buffer. Total RNA was purified using the NucleoSpin RNA Plus kit (Macherey-Nagel, Germany) according to the manufacturer’s instructions. The procedure was repeated to obtain 3 biological replicates of total RNA. Three micrograms of each sample were treated with a Turbo DNA-free kit (Invitrogen, USA) to eliminate genomic DNA. Reverse transcription was carried out using 100 ng of each RNA sample as a template using the High-Capacity cDNA Reverse transcription kit (Applied Biosystems, United States) according to the manufacturer’s instructions.

All cDNA samples were amplified using gene-specific qRT-PCR primers (Table S2) and SYBR Green PCR reagent (Qiagen, Germany) on a Rotor-Gene 6000 real-time thermocycler. The thermocycler profile included 5 min of initial denaturation at 95°C and 40 cycles of denaturation (95°C for 5 s) and annealing/extension (60°C for 10 s). Reactions were carried out in 2 technical replicates for 3 biological replicates. The CT values of all reactions were obtained using the Rotor-Gene Q Series Software. The internal reference gene *rpoB* was used. Using the 2^-ΔCT^ method, the relative expression levels of the target gene were normalized to the internal reference gene.
