# Supplementary material for: A Novel Plasmid Entry Exclusion System in pKPC_UVA01, a Promiscuous Conjugative Plasmid Carrying the blaKPC Carbapenemase Gene
Source: Antimicrob Agents Chemother. 2022 Mar 15;66(3):e02322-21. doi: 10.1128/aac.02322-21 (PMC8923210; doi:10.1128/aac.02322-21)
Supplement: Supplemental file 1 — Tables S1 and S2 and Fig. S1 to S4. Download aac.02322-21-s0001.pdf, PDF file, 0.3 MB [file aac.02322-21-s0001.pdf]

Supplementary materials for

A novel plasmid entry exclusion system in pKPC_UVA01, a promiscuous conjugative plasmid carrying the *bla*$_{KPC}$ carbapenemase gene

Muhammad Kamruzzaman[a#], Amy J Mathers[b], Jonathan R Iredell[a,c#]

[a] Centre for Infectious Diseases and Microbiology, The Westmead Institute for Medical Research, The University of Sydney, Westmead, New South Wales, Australia.

[b] Division of Infectious Disease and International Health, Department of Medicine, University of Virginia Health System, Charlottesville, VA, USA; Clinical Microbiology Laboratory, Department of Pathology, University of Virginia Health System, Charlottesville, VA, USA.

[c] Westmead Hospital, Westmead, New South Wales, Australia.

# Address correspondence to muhammad.kamruzzaman@sydney.edu.au or jonathan.iredell@sydney.edu.au

Table S1. Characteristics of plasmids having the novel *trbK* entry exclusion gene

Plasmid	Accession number	Plasmid Inc type	Size (kb)	Major resistance genes
K. pneumoniae plasmid pKPC_UVA01	CP017937.1	pKPC_UVA01 type	43	*bla*$_{TEM}$, *bla*$_{KPC-2}$
C. freundii plasmid pKPC_CAV1857-43	CP037739.1	pKPC_UVA01 type	43	*bla*$_{TEM}$, *bla*$_{KPC-2}$
C. freundii complex sp. plasmid pKPC-9d88	CP026239.1	pKPC_UVA01 type	53	*bla*$_{TEM}$, *bla*$_{KPC-2}$
K. pneumoniae strain AR_0126 plasmid tig00000003	CP021743.1	pKPC_UVA01 type	43	*bla*$_{TEM}$, *bla*$_{KPC-2}$
K. pneumoniae plasmid pKPC_CAV1193	CP013325.1	pKPC_UVA01 type	49	*bla*$_{TEM}$, *bla*$_{KPC-2}$
C. freundii plasmid pKPC_CAV1741	CP011656.1	IncM1/ pKPC_UVA01 type	129	*bla*$_{TEM}$, *bla*$_{KPC-2,}$ *bla*$_{SHV-30,}$ *aac(6')-Ib-cr*
K. pneumoniae plasmid pCAV1344-40	CP011620.1	pKPC_UVA01 type	39	*bla*$_{TEM}$
C. freundii plasmid pKPC_CAV1321-45	CP011608.1	pKPC_UVA01 type	45	*bla*$_{TEM}$, *bla*$_{KPC-2}$
E. hormaechei plasmid pKPC_CAV1668	CP011582.1	pKPC_UVA01 type	43	*bla*$_{TEM}$, *bla*$_{KPC-2}$
K. pneumoniae plasmid pKPC_CAV1392	CP011575.1	pKPC_UVA01 type	43	*bla*$_{TEM}$, *bla*$_{KPC-3}$
E. hormaechei plasmid pCAV1311-34	CP011570.1	pKPC_UVA01 type	33	*bla*$_{TEM}$
E. hormaechei plasmid p34983-43.621kb	CP010379.1	pKPC_UVA01 type	43	*bla*$_{TEM}$, *bla*$_{KPC-2}$
E. hormaechei plasmid p34977-43.621kb	CP010374.1	pKPC_UVA01 type	43	*bla*$_{TEM}$, *bla*$_{KPC-2}$
C. freundii plasmid p1C44	CP054297.1	pKPC_UVA01 type	43	*bla*$_{TEM}$, *bla*$_{KPC-2}$
K. pneumoniae plasmid p2K44	CP054293.1	pKPC_UVA01 type	43	*bla*$_{TEM}$, *bla*$_{KPC-2}$
K. pneumoniae plasmid p3K44	CP054288.1	pKPC_UVA01 type	43	*bla*$_{TEM}$, *bla*$_{KPC-2}$
E. coli plasmid p5E44	CP054283.1	pKPC_UVA01 type	43	*bla*$_{TEM}$, *bla*$_{KPC-2}$
C. koseri genome assembly PRJEB6512_assembly_1	LK931338.1	pKPC_UVA01 type	33	None
L. adecarboxylata plasmid p16005813B	MK036884.1	pKPC_UVA01 type	45	*bla*$_{IMP-8,}$ *aac(6')-Ib-cr*
K. pneumoniae strain AR_0129 plasmid tig00000003	CP021716.1	pKPC_UVA01 type	43	*bla*$_{TEM}$, *bla*$_{KPC-3}$
E. coli strain plasmid pECAZ161_KPC	CP019010.1	ColRNAI/ pKPC_UVA01 type	55	*bla*$_{TEM}$, *bla*$_{KPC-3}$
K. pneumoniae plasmid pKPC_Kp46	KX348146.1	pKPC_UVA01 type	49	*bla*$_{TEM}$, *bla*$_{KPC-3,}$ *bla*$_{OXA-9,}$ *aac(6')-Ib-cr*
S. marcescens plasmid pSmUNAM836	CP012686.1	pKPC_UVA01 type	26	*bla*$_{TEM}$
E. cloacae plasmid p35734-109.753kb	CP012163.1	pKPC_UVA01 type	109	*bla*$_{TEM}$, *bla*$_{KPC-3,}$ *bla*$_{OXA-9,}$ *aac(6')-Ib-cr*
E. hormaechei plasmid p34399-43.500kb	CP010387.1	pKPC_UVA01 type	43	*bla*$_{TEM}$, *bla*$_{KPC-2}$
K. pneumoniae plasmid pCRE-195-5	CP061394.1	pKPC_UVA01 type	43	*bla*$_{TEM}$, *bla*$_{KPC-2}$

K. quasipneumoniae plasmid pKp4101	CP047282.1	IncC/ pKPC_UVA01 type	187	*bla*$_{TEM}$, *bla*$_{CMY-4}$, *bla*$_{OXA-204}$, *aac(6')-Ib-cr*
E. hormaechei plasmid pKPC_49790_VIM_1	CP059426.1	pKPC_UVA01 type	55	*bla*$_{TEM}$, *bla*$_{VIM-1}$, *qnrS1*, *aac(6')-Ib-cr*
E. hormaechei plasmid pKPC_48212_VIM	CP059417.1	pKPC_UVA01 type	55	*bla*$_{TEM}$, *bla*$_{VIM-1}$, *qnrS1*, *aac(6')-Ib-cr*
K. pneumoniae ABFQB plasmid pKPC-e4b7	CP036440.1	pKPC_UVA01 type	51	*bla*$_{KPC-3}$, *qnrS1*
K. pneumoniae KPNIH45 plasmid pKPC-e4b7	CP036448.1	pKPC_UVA01 type	51	*bla*$_{KPC-3}$, *qnrS1*
C. freundii plasmid pKPC-e4b7	CP036437.1	pKPC_UVA01 type	51	*bla*$_{KPC-3}$, *qnrS1*
E. hormaechei plasmid unnamed3	CP027143.1	pKPC_UVA01 type	52	*bla*$_{KPC-2}$
K. pneumoniae plasmid pMNCRE53_3	CP018436.1	pKPC_UVA01 type	52	*bla*$_{KPC\ -2}$, *qnrS1*
K. pneumoniae plasmid pMNCRE78_3	CP018432.1	pKPC_UVA01 type	52	*bla*$_{KPC-2}$, *qnrS1*
K. pneumoniae plasmid pMNCRE69_3	CP018426.1	pKPC_UVA01 type	52	*bla*$_{KPC-2}$, *qnrS1*
K. pneumoniae plasmid pKPC_CAV1042-44	CP018668.1	pKPC_UVA01 type	43	*bla*$_{TEM}$, *bla*$_{KPC-2}$

Table S2. Primers used in this study

Primer	Sequence(5′-3′)*	Reference/ Accession number
RepA1_F	ATGGACTGATCAAGCTGGCG	This study; CP009465
RepA1_R	TTTGACGCGGCCATATACGG	This study; CP009465
TrbK_F_HindIII	CGAAGCTTCAGCAAGCGCAGACTAATACCG	This study; CP009465
TrbK_R_XbaI	CGTCTAGAGCAAACATGCCAAACAGAAA	This study; CP009465
TrbK_116CT_R_XbaI	CGTCTAGACCGGCCATCTTATTGCTGTA	This study; CP009465
TrbK_232CT_R_XbaI	CGTCTAGACGAGGGGTTCCACGATTAGG	This study; CP009465
TrbJ_F_HindIII	CGAAGCTTGCGTTCGGTATTTCTCCTGA	This study; CP009465
TrbJ_R_XbaI	CGTCTAGATTATTTATCCCCTTTATATGAATCAGC	This study; CP009465
ATPase_F_HindIII	CGAAGCTTCCGCTTGCGTAGCAATCCTT	This study; CP009465
TrbK_FRT_P1	CGTTGCATTAAATATTGTCTCTTTAAGTGCTAATGCGGC**GTGTAGGCTGGAGCTGCTTC**	This study; CP009465
TrbK_FRT_P2	CAAGATGCCTCAAAATCAACATTTGCTTGTGTTATTTTCC**CATATGAATATCCTCCTTA**	This study; CP009465
TrbJ_FRT_P1	TGGCTGGTATCCTTTCCTCTCCTGTTTATGCAGGTGTGCC**GTGTAGGCTGGAGCTGCTTC**	This study; CP009465
TrbJ_FRT_P2	CTGACGCGCAATTAATTGTTGAGTTGCAAGCGAATCATTG**CATATGAATATCCTCCTTA**	This study; CP009465
TrbL_FRT_P1	GCCGTGCTTTCAAGTTCGGGTAGTGACATTTATGTGTTCG**GTGTAGGCTGGAGCTGCTTC**	This study; CP009465
TrbL_FRT_P2	CCCGCAGCATTGATTGTCGCTTTCATCACCAACATGGT**CATATGAATATCCTCCTTA**	This study; CP009465
TrbL_F	GTTATGGCGGTGGCGACATT	This study; CP009465
TrbL_R	TCACCCGCAGCATTGATTGT	This study; CP009465
TrbL_HindII_F	CGAAGCTTGGCCGGGGAAAATAACACA	This study; CP009465
TrbL_XbaI_R	CGTCTAGACCGAGCGCAGAACAGCAAAT	This study; CP009465
AbR_nest_F1	GCCAATATCACGTTAGCCACGTCAGAAAGCTGGCGTGAC**ATAGAGCGGGGATTAGTGTGG**	This study; CP009465
AbR_nest_R1	GCCAGCTTGATCAGTCCATTTACGTGTTCTGAGCTGGCC**GCTGTGGATCTGCACGTTGAA**	This study; CP009465
AbR_nest_F2	AAGATCCTGAAGTGCGTTATATGCCGAACGGCGGCGCAGTT**GCCAATATCACGTTAGCCA**	This study; CP009465
AbR_nest_R2	CACCGACATTCACAACAACTTCTGTGGTGTATTTCTCCAC**GCCAGCTTGATCAGTCCATT**	This study; CP009465
TrbK_F_EcoRI	GCGAATTCGAGCAAGAGCGCTTAAAGAAA	This study; CP009465
trbK_F_RT	TTGATGAGCAGCATAAGCCACTG	This study; CP009465
trbK_R_RT	CGAGGGGTTCCACGATTAGG	This study; CP009465

* Restriction enzyme cutting sites are underlined, **bold** nucleotide are for gene-specific sequences and plain nucleotides with bold characters are homologous arm from the deletion target region.

Figure S1

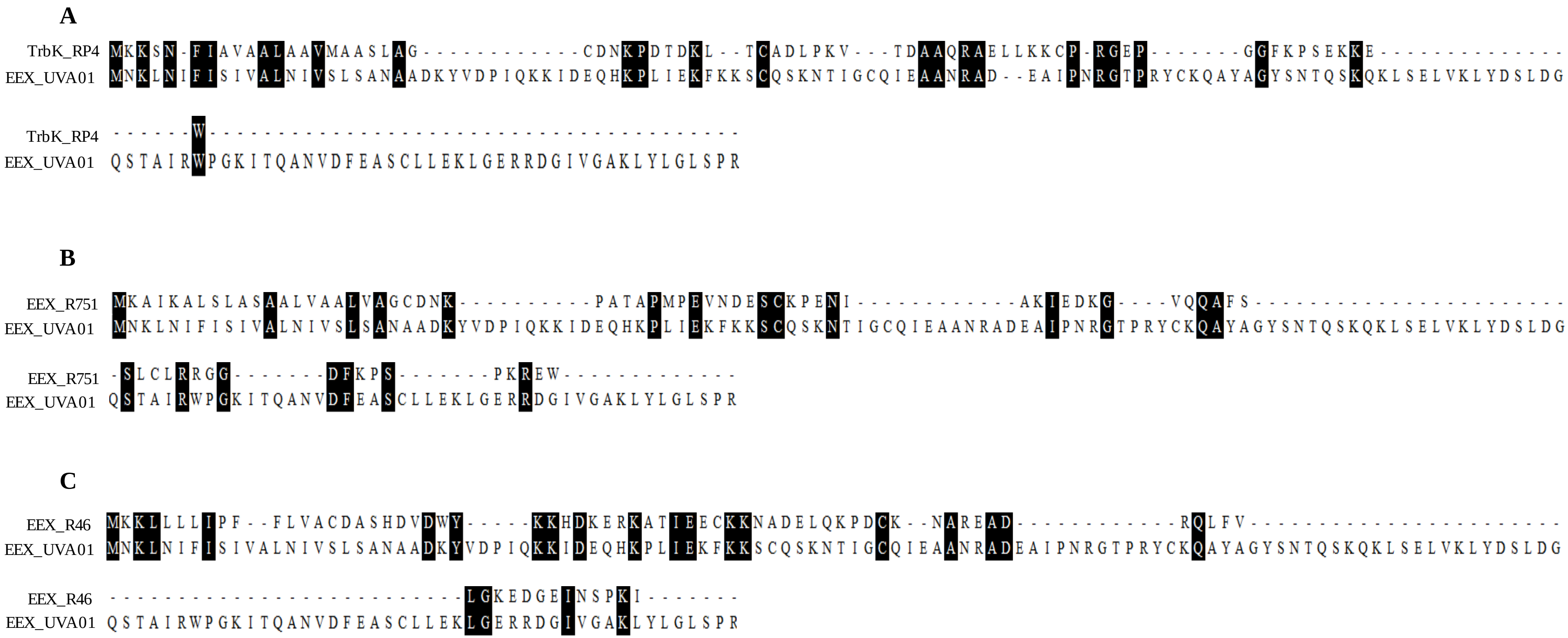

Figure S1. Amino acid sequence alignment of putative entry exclusion protein of pKPC_UVA01 with that of IncP plasmid RP4 (A), IncP plasmid R751 (B) and IncN plasmid R46 (C). Identical amino acids are shaded in black.

1 CGAAGCTTCAGCAAGCGCAGACTAATACCGACTTTAGTATGTCTAATCAAATTGCTAATG 60

61 ATTATTTTAATAAGATGGCAAGAGCAAGAGCGCTTAAAGAAAAGTCTATGGCTGATTCAT 120

121 ATAAAGGGGATAAATAATGAATAAGTTAAATATATTTATTTCAATCGTTGCATTAAATAT 180

181 TGTCTCTTTAAGTGCTAATGCGGCTGATAAATATGTTGATCCAATTCAAAAGAAAATTGA 240

241 TGAGCAGCATAAGCCACTGATTGAAAAATTCAAAAAAAGCTGTCAATCAAAAAACACGAT 300

301 AGGATGTCAGATAGAGGCCGCTAATCGCGCTGATGAAGCGATTCCTAATCGTGGAACCCC 360

361 TCGTTATTGTAAGCAAGCCTATGCTGGTTACTCTAATACACAGAGTAAACAAAAGCTAAG 420

421 TGAGTTAGTTAAGCTATATGACTCTCTTGATGGACAGTCTACAGCAATAAGA**TAA**CCGGG 480

481 GAAAATAACACAAGCAAATGTTGATTTTGAGGCATCTTGCTTGCTCGAAAAACTTGGCGA 540

541 ACGTAGAGACGGTATTGTAGGCGCAAAATTATATCTTGGGTTATCCCCTAGGTAAATAAT 600

601 TAGGCGTTTTTTTATTACGGTTAGTCCGTAAGGAGATTACTTTGTCTAAAAACAGGCTTT 660

661 TGATATTTCTGTTTGGCATGTTTGCTCTAGACG

Fig. S2. Nucleotide sequence synthesized with an early termination codon at the C-terminal region of *trbK*. HindIII and XbaI restriction sites are underlined. The early termination codon is shown in red-bold characters (GG>AA changes). *trbK* coding region is shaded grey.

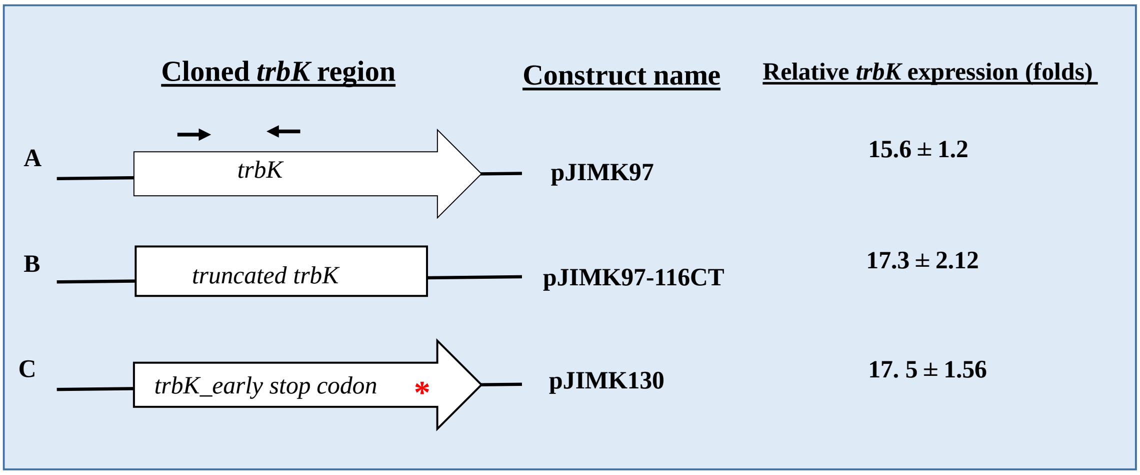

Fig. S3. Relative *trbK* expression from different *trbK* constructs in *E.coli*. The RT-qPCR primers position were indicated by two forward and reverse facing arrows. Relative expression values are the mean of 3 biological replicates with standard errors. The red asterics in pJIMK130 indicates the position of the early termination codon.

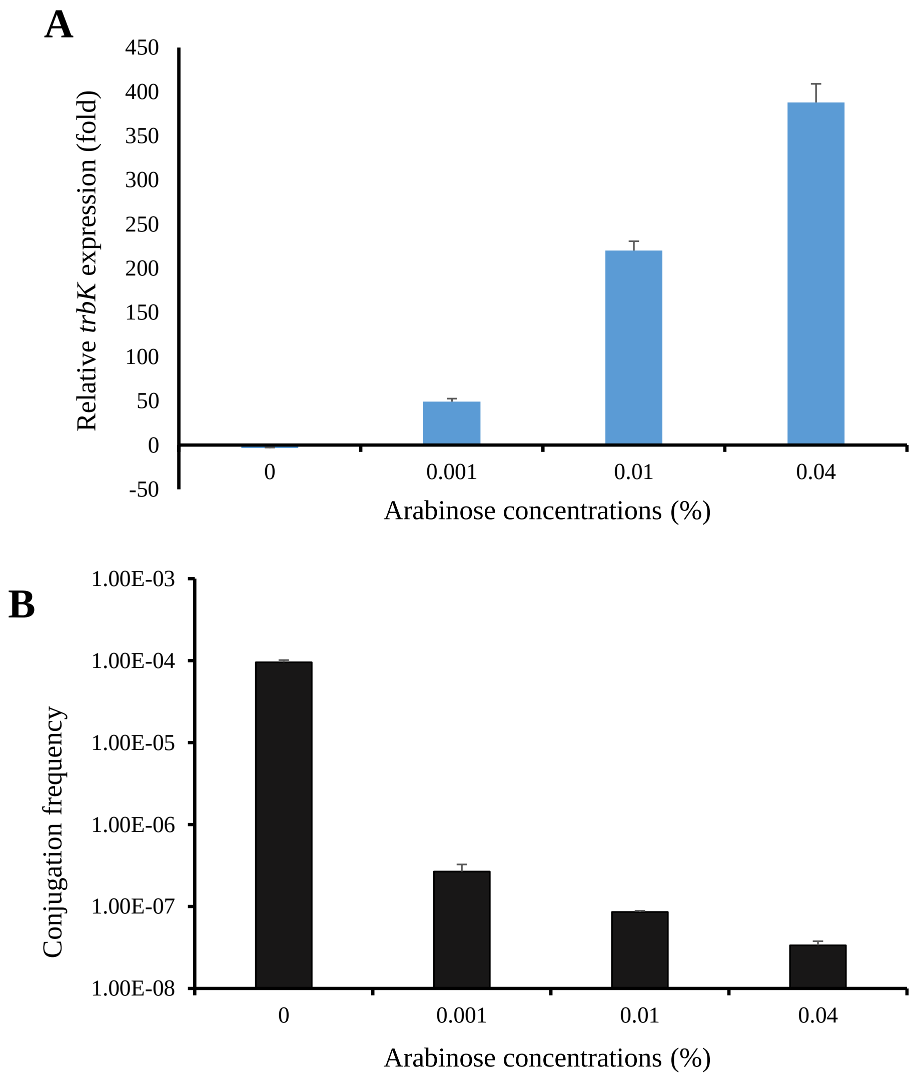

Fig. S4. Relative *trbK* expression from BW25113Rf(pJIMK131) after induction with different dosages of arabinose (A). Conjugation transfer frequence of pKPC_UVA01 plasmid from J53 to recipient BW25113Rf(pJIMK131) after induction with different dosages of arabinose (B). The results are the mean of 3 independent experiments with standard errors.
